# Supplementary material for: The Role of Monk Parakeets as Nest-Site Facilitators in Their Native and Invaded Areas
Source: Biology (Basel). 2021 Jul 19;10(7):683. doi: 10.3390/biology10070683 (PMC8301312; doi:10.3390/biology10070683)
Supplement: Supplementary file 1 [file biology-10-00683-s001.zip › FigureS1.pdf]

Model fits

Diagnostic tools provided by the DHARMa package in R [71] were used to evaluate the fit of the final models. DHARMa simulates quantile residuals from a fitted GLM that are standardized to values between 0 and 1. For a correctly specified model, these residuals should have a uniform distribution regardless of the underlying model structure and can be interpreted similarly to residuals for linear models. The package includes statistical tests on the residuals to check for uniformity, overdispersion, and zero inflation.

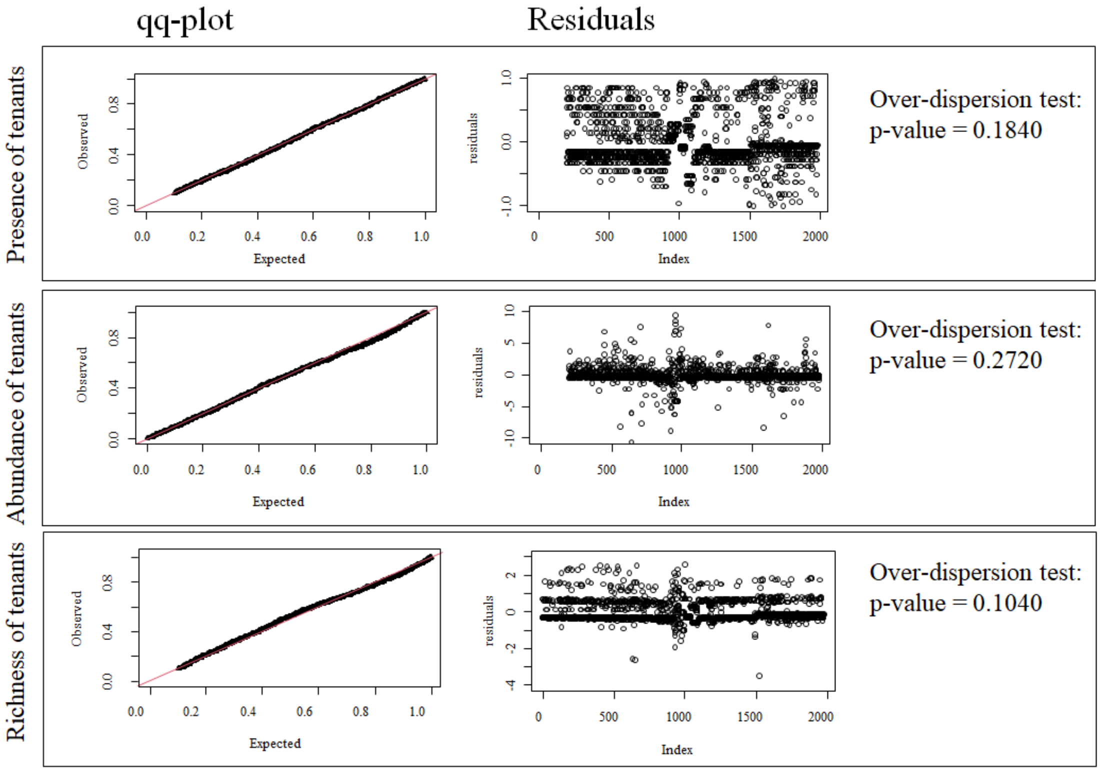

Figure S1. Non-parametric dispersion test and standard residuals plots for the final models obtained to assess the effects of nest size (i.e., number of chambers), abandonment, habitat type, substrate, height, and population (South America, Puerto Rico, Spain, and Tenerife) on the presence, abundance, and richness of tenant species at monk parakeet nests. No significant problems were detected.
