# Supplementary material for: The Role of Monk Parakeets as Nest-Site Facilitators in Their Native and Invaded Areas
Source: Biology (Basel). 2021 Jul 19;10(7):683. doi: 10.3390/biology10070683 (PMC8301312; doi:10.3390/biology10070683)
Supplement: Supplementary file 1 [file biology-10-00683-s001.zip › FigureS2.pdf]

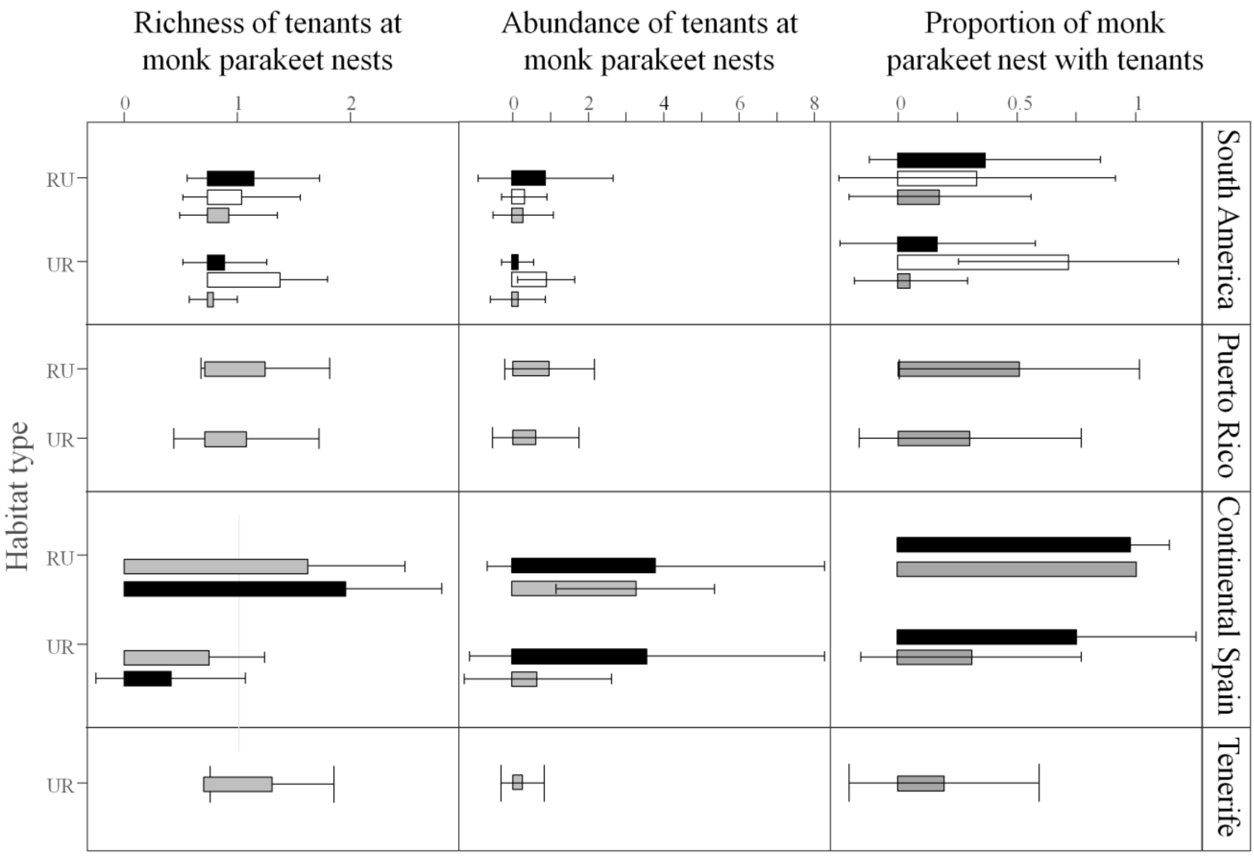

Figure S2. Proportion of nests (±SD) with tenants, and abundance (±SD) and richness (±SD) of tenants in populations of monk parakeets *Myiopsitta monachus* from South America (Argentina, Brazil, Paraguay, and Uruguay), Puerto Rico, Continental Spain, and Tenerife. Bars represent nests located in pylons (black bars), roofs (white bars) and trees (grey bars), grouped by urban (UR) and rural (RU) habitats.
