# Supplementary material for: The Role of Monk Parakeets as Nest-Site Facilitators in Their Native and Invaded Areas
Source: Biology (Basel). 2021 Jul 19;10(7):683. doi: 10.3390/biology10070683 (PMC8301312; doi:10.3390/biology10070683)
Supplement: Supplementary file 1 [file biology-10-00683-s001.zip › TableS1.pdf]

Table S1. Post-hoc test (including Bonferroni's correction) comparing the proportion of monk parakeets with tenant species, as well as its abundance and richness in the different monitored populations.

**Presence of tenants**					
**contrast**	**odds.ratio**	**SE**	**df**	**t-test**	**p**
Puerto Rico/South America	21.72	6.86	1965	9.74	<0.0001
C. Spain/South America	51.53	16.24	1965	12.51	<0.0001
C. Spain/Puerto Rico	2.37	0.77	1965	2.67	0.0463
Tenerife/South America	17.45	9.09	1965	5.49	<0.0001
Tenerife/Puerto Rico	0.80	0.43	1965	-0.41	1.0000
Tenerife/C. Spain	0.34	0.14	1965	-2.55	0.0657
**Abundance of tenants**					
**contrast**	**ratio**	**SE**	**df**	**t-test**	**p**
Puerto Rico/South America	6.05	1.14	1962	9.58	<0.0001
C. Spain/South America	5.33	0.82	1962	10.83	<0.0001
C. Spain/Puerto Rico	0.88	0.16	1962	-0.70	1.0000
Tenerife/South America	1.84	0.71	1962	1.59	0.6759
Tenerife/Puerto Rico	0.30	0.12	1962	-3.01	0.0157
Tenerife/C. Spain	0.35	0.12	1962	-3.02	0.0153
**Richness of tenants**					
**contrast**	**ratio**	**SE**	**df**	**t-test**	**p**
Puerto Rico/South America	3.34	0.59	1963	6.84	<0.0001
C. Spain/South America	4.19	0.62	1963	9.71	<0.0001
C. Spain/Puerto Rico	1.26	0.22	1963	1.31	1.0000
Tenerife/South America	6.01	1.45	1963	7.42	<0.0001
Tenerife/Puerto Rico	1.80	0.46	1963	2.30	0.1296
Tenerife/C. Spain	1.43	0.27	1963	1.89	0.3515
