# Supplementary material for: The Role of Monk Parakeets as Nest-Site Facilitators in Their Native and Invaded Areas
Source: Biology (Basel). 2021 Jul 19;10(7):683. doi: 10.3390/biology10070683 (PMC8301312; doi:10.3390/biology10070683)
Supplement: Supplementary file 1 [file biology-10-00683-s001.zip › TableS2.pdf]

Table S2. Alternative models (i.e., models ΔAICc < 2) obtained to compare the proportion of monk parakeets with tenant species, the abundance and richness of tenants, as well as the persistence (i.e., years that a nest remained occupied) of tenants at monk parakeet nests.

**Presence of tenants**					
**Models**	**Variables**	**Estimate**	**SE**	**z value**	**p**
A + B + C + D + E	(Intercept)	-1.82027	0.32718	-5.563	2.64e-08
	A	22.61738	2903.28357	0.008	0.9938
	B	1.59162	0.09466	16.813	< 2e-16
	C (urban)	-1.39399	0.33566	-4.153	3.28e-05
	D (Puerto Rico)	3.13453	0.32259	9.717	< 2e-16
	D (C. Spain)	2.94282	0.36235	8.121	4.60e-16
	D (Tenerife)	1.26833	0.63091	2.01	0.0444
	E (Roof)	3.88229	0.66525	5.836	5.35e-09
	E (Tree)	-0.66036	0.34939	-1.89	0.0588
A + B + C + D + E + F	(Intercept)	-1.83075	0.32964	-5.554	2.79e-08
	A	23.16831	3826.98956	0.006	0.9952
	B	1.58862	0.09524	16.681	< 2e-16
	C (urban)	-1.39713	0.33603	-4.158	3.21e-05
	F	0.01787	0.06688	0.267	0.7894
	D (Puerto Rico)	3.14835	0.32681	9.634	< 2e-16
	D (C. Spain)	2.94534	0.36261	8.123	4.56e-16
	D (Tenerife)	1.2772	0.63191	2.021	0.0433
	E (Roof)	3.91614	0.67751	5.78	7.46e-09
	E (Tree)	-0.65005	0.35152	-1.849	0.0644
**Abundance of tenants**					
**Models**	**Variables**	**Estimate**	**SE**	**z value**	**p**
A + B + C + D + E	(Intercept)	-0.93586	0.15224	-6.147	7.88e-10
	A	1.54357	0.13083	11.798	< 2e-16
	B	0.86922	0.04021	21.618	< 2e-16
	C (urban)	-0.97081	0.14168	-6.852	7.27e-12
	D (Puerto Rico)	1.80033	0.188	9.576	< 2e-16
	D (C. Spain)	1.67379	0.15455	10.83	< 2e-16

	D (Tenerife)	0.61023	0.38449	1.587	0.112
	E (Roof)	1.39809	0.35578	3.93	8.51e-05
	E (Tree)	-0.68237	0.15631	-4.365	1.27e-05
A + B + C + F + D + E	(Intercept)	-0.94112	0.15248	-6.172	6.74e-10
	A	1.53552	0.132	11.633	< 2e-16
	B	0.86718	0.04033	21.504	< 2e-16
	C (urban)	-0.96275	0.14275	-6.744	1.54e-11
	F	0.01695	0.04033	0.42	0.674
	D (Puerto Rico)	1.80999	0.18917	9.568	< 2e-16
	D (C. Spain)	1.66621	0.15534	10.726	< 2e-16
	D (Tenerife)	0.60911	0.38417	1.586	0.113
	E (Roof)	1.41671	0.35792	3.958	7.55e-05
	E (Tree)	-0.6777	0.15638	-4.334	1.47e-05

**Richness of tenants**					
**Models**	**Variables**	**Estimate**	**SE**	**z value**	**p**
A + B + C + D + E	(Intercept)	-1.76624	0.14849	-11.894	< 2e-16
	A	1.07473	0.11225	9.574	< 2e-16
	B	0.32126	0.01639	19.598	< 2e-16
	C (urban)	-0.85258	0.12596	-6.769	1.30e-11
	D (Puerto Rico)	1.20487	0.17611	6.841	7.84e-12
	D (C. Spain)	1.43272	0.14757	9.709	< 2e-16
	D (Tenerife)	1.79366	0.2417	7.421	1.16e-13
	E (Roof)	1.93722	0.29746	6.513	7.39e-11
	E (Tree)	0.18719	0.13433	1.394	0.163
A + B + C + F + D + E	(Intercept)	-1.766265	0.148498	-11.894	< 2e-16
	A	1.075947	0.113035	9.519	< 2e-16
	B	0.321217	0.016396	19.591	< 2e-16
	C (urban)	-0.854079	0.127033	-6.723	1.78e-11
	F	-0.003371	0.036853	-0.091	0.927
	D (Puerto Rico)	1.20268	0.177713	6.768	1.31e-11
	D (C. Spain)	1.434358	0.148655	9.649	< 2e-16

	D (Tenerife)	1.79405	0.241755	7.421	1.16e-13
	E (Roof)	1.934079	0.299391	6.46	1.05e-10
	E (Tree)	0.187236	0.134338	1.394	0.163

**Persistence of tenants**					
**Models**	**Variables**	**Estimate**	**SE**	**z value**	**p**
G + A + B + C + F + D + E	(Intercept)	4.23816	1.10091	3.85	0.000118
	G	-8.06706	0.4879	-16.534	< 2e-16
	A	2.05852	0.56596	3.637	0.000276
	B	9.9667	0.53242	18.72	< 2e-16
	C (urban)	-5.83022	1.6697	-3.492	0.00048
	F	-0.43353	0.08053	-5.383	7.31e-08
	D (Seville)	0.19793	0.15204	1.302	0.192985
	D (Tenerife)	0.16061	0.22823	0.704	0.48159
	E (Tree)	0.92755	1.44476	0.642	0.520869
G + A + B + C + F + D	(Intercept)	4.40145	1.07191	4.106	4.02e-05
	G	-8.07019	0.48789	-16.541	< 2e-16
	A	2.01843	0.55797	3.617	0.000298
	B	9.96988	0.53242	18.726	< 2e-16
	C (urban)	-5.06737	1.0721	-4.727	2.28e-06
	F	-0.43285	0.08045	-5.38	7.43e-08
	D (Seville)	0.2004	0.15194	1.319	0.187196
	D (Tenerife)	0.163	0.22816	0.714	0.474972

A: Abandoned nest (i.e., without presence of monk parakeets)
B: nest size (i.e., number of chambers)
C: habitat type (urban/rural)
D: population (South America, Puerto Rico, Continental Spain, and Tenerife) or (Seville, Madrid, and Tenerife)
E: substrate (tree, pylon, roof)
F: height above ground level
G: number of monk parakeets in the colony
